# BerTime: A novel tool for supporting ALS algorithm application in clinical practice

**DOI:** 10.1016/j.resplu.2024.100636

**Published:** 2024-04-25

**Authors:** Bertuol Maria, Guasconi Massimo, Bonacaro Antonio, Stirparo Giuseppe, Bignami Elena Giovanna

**Affiliations:** Department of Medicine and Surgery, University of Parma, Parma, Italy; Department of Medicine and Surgery, University of Parma, Azienda USL di Piacenza, Piacenza, Italy; Department of Medicine and Surgery, University of Parma, Parma, Italy; Società Italiana di Medicina e Divulgazione Scientifica (SIMED), Parma, Italy; Department of Medicine and Surgery, University of Parma, Parma, Italy

This contribution aims at proposing a new tool (Table 1) useful for improving the accuracy of Advanced Life Support (ALS) manoeuvres, in full accordance with the American Heart Association 2020 guidelines^1^ and with the European Resuscitation Council 2021 ones.^2^ The goal is to provide healthcare professionals and educators with a comprehensive tool designed to support, monitor, check, and implement the quality of the aforementioned procedures. The proposed novel tool is named BerTime.

**Table 1:** BerTime: a novel tool designed in accordance with AHA and ERC ALS guidelines.

The importance of adhering to the ALS algorithm is fundamental for maximising the rate of patients’ survival; literature shows a variety of tools and medical devices capable of providing immediate audio-visual feedback to emergency teams. These are essential for assessing the quality of chest compressions and lung ventilation.^3^ However, only a few tools may also act as a reporting system; some advanced monitor defibrillators fulfil this need by recording the beginning of the manoeuvres and the various actions performed by emergency teams such as drug administration, intubation, etc. This feature is very useful, but not everyone has access to these types of monitors, and not all kinds of professionals are able to use them. Volunteers, Basic Life Support and Defibrillation trained citizens, and bystanders guided over the phone with pre-arrival instructions can, in case of Sudden Cardiac Death (SCA), proceed to use the Automatic External Defibrillator (AED). Therefore, this medical device does not possess these characteristics. Therefore, the proposed tool attempts to reconcile the multiple salient facets of the algorithm from a clinical, educational, and quality assurance perspective. It allows to:

– enhancing the monitoring of procedural developments, maintaining awareness of rapidly occurring cycles, and providing support to junior staff members during their training.
– promptly recording any performed action (team member performing ventilations will use the tool, while the other one is performing chest compression).

Furthermore, the newly designed tool allows to:

– tracking number of cycles: numbers are arranged in rows of 5, if chest compressions and ventilations are delivered on time, rhythm analysis will follow every 2 minutes. According to the international guidelines^1^, ^2^ a team member is designated to ensure the correct application of the algorithm including the appropriate counting
– tracking number of delivered shocks (rhythm analysis column)
– documenting reversible causes^1^
– documenting drug/shock administration (adrenaline column, white boxes for non-shockable rhythm, and green boxes for shockable ones)^1^
– recording the time when the manoeuvres start (first row), any sign of Return of Spontaneous Circulation (ROSC time column), and the time when manoeuvres end (last row)
– producing a full report of the intervention usable for debriefing^4^ and for handover
– monitoring and recording possible errors or criticalities, even in a simulated clinical environment for training purposes.^5^

This novel tool is extremely versatile and may be implemented in both paper and digital formats.

The last two columns of the tool are foldable, so they can be hidden from the layperson, limiting the number of information shown to the strictly necessary.

## Declaration of competing interest

The authors declare that they have no known competing financial interests or personal relationships that could have appeared to influence the work reported in this paper.
